# Factors Influencing Engagement in Work‐Related Activities Among People With Dementia or Mild Cognitive Impairment: A Cross‐Sectional Study

**DOI:** 10.1155/oti/5966517

**Published:** 2026-04-18

**Authors:** Erika Kamo, Yuma Sonoda, Takuma Yuri, Kayano Yotsumoto, Hisatomo Kowa

**Affiliations:** ^1^ Department of Rehabilitation Science, Kobe University Graduate School of Health Sciences, Kobe, Hyogo, Japan, kobe-u.ac.jp; ^2^ Department of Occupational Therapy, Kyoto Tachibana University, Kyoto, Japan, tachibana-u.ac.jp

## Abstract

**Introduction:**

People with dementia or mild cognitive impairment (MCI) desire social connection and meaningful contribution, despite often being excluded from work‐related activities. The impact of engagement in work‐related activities on the mental well‐being of people with dementia or MCI remains underexplored.

**Objective:**

The aim of this study is to investigate the factors influencing engagement in work‐related activities among people with dementia or MCI.

**Design:**

Cross‐sectional study.

**Setting:**

Seven‐day care centers in Japan.

**Participants:**

Sixty‐three day care center members with dementia or MCI who had participated in work‐related activities for at least 6 months.

**Measures:**

Well‐being assessed by the World Health Organization‐Five Well‐Being Index (WHO‐5); engagement evaluated by the Assessment of Quality of Activities (A‐QOA), an occupational therapists–developed observational assessment tool; and confounders of engagement.

**Results:**

Among the 63 participants (age: median: 83 years, interquartile range [IQR]: 61, 94; Mini‐Mental State Examination‐Japanese score: median: 19 points, IQR: 4, 27), 74.6% were female. A significant correlation was observed between the WHO‐5 and A‐QOA (*ρ* = 0.433, *p* < 0.001). Stepwise multiple regression analysis revealed a significant association between the A‐QOA and WHO‐5 (*β* = 0.480, *p* < 0.001), frequency of participation (*β* = 0.337, *p* = 0.003), and hearing loss–related social limitations (*β* = 0.286, *p* = 0.01).

**Conclusion:**

Greater engagement in work‐related activities was associated with better well‐being, higher frequency of participation, and hearing loss–related social limitations. Implementation of and support for personalized work‐related activities, considering these factors for people with dementia or MCI, could enhance their engagement and promote their mental well‐being.

## 1. Introduction

Globally, the incidence of dementia is increasing exponentially [1]. Japan has one of the most rapidly aging societies [2] with approximately 4.4 million people with dementia and 10 million people with dementia or mild cognitive impairment (MCI) [3]. The recently introduced lecanemab and other new drugs for dementia are promising pharmacological therapies. However, their effect is limited to inhibiting progression, and their side effects or incompatibility with underlying pathologies preclude their prescription for many people with dementia [4]. Therefore, the role of nonpharmacological therapies becomes more important for people with dementia or MCI.

Community‐dwelling people with dementia often desire continuing social contact, enhancing self‐esteem, being useful, and participating in enjoyable and meaningful activities [5, 6]. People with MCI experience challenges in social connections and participation [7]. Additionally, cognitive impairments and psychiatric symptoms such as depression weaken their mental well‐being [5, 8]. Nonpharmacological therapies that enhance the mental well‐being of people with dementia include social interaction, meaningful contribution [9], and exercise [10]. Work‐related activities that combine these elements can help maintain and enhance the mental well‐being of people with dementia or MCI.

Some people with dementia yearn to “connect with society and contribute meaningfully” [11, 12]. In Japan, as a practical response to such voices, older people with specific care needs, including those with dementia or MCI, under long‐term care insurance, can conditionally participate in work‐related activities [13]. For example, people with dementia or MCI have been participating in community cleaning, car washing, assisting staff at convenience stores and cafes, and so on, depending on the individual community circumstances and facilitated by multiagency collaborations.

Qualitative research suggests that work‐related activities may improve the mental well‐being of people with dementia or MCI [14, 15]. Intervention studies have reported significantly improved mental well‐being [16, 17], while others have reported no change in depression or sense of purpose and usefulness [18] among people with dementia or MCI who participated in work‐related activities. A recent multicenter cross‐sectional study in Japan also reported that engagement in work‐related activities was associated with mental well‐being in this population [19]. In intervention studies, a clear consensus has not been reached regarding the influence of work‐related activities on mental well‐being.

Understanding how work‐related activities influence mental well‐being may help develop effective methods to improve the mental well‐being of people with dementia through such activities.

Ura et al. [17] and George’s [18] interview‐ and observation‐based qualitative evaluations of people with dementia who participated in work‐related activities found that how individuals were occupied or involved in the activity differed depending on participants′ prior experiences and other related factors. Conceptually, “the act of being occupied or involved with an external stimulus” is defined as “engagement” [20]. Jones et al. [21] found that engagement promotes well‐being by giving people opportunities to use their skills, a sense of belonging, meaning in life, and encouragement to feel good about themselves. Therefore, engagement through participation in work‐related activities may be related to improvement of mental well‐being.

In this study, we aimed to investigate the factors influencing engagement in work‐related activities among people with dementia or MCI. Furthermore, factors related to engagement in work‐related activities from the perspective of the individual, stimulus, and environment [22], which reportedly influence engagement in people with dementia, were investigated.

## 2. Materials and Methods

### 2.1. Study Design

This multicenter cross‐sectional study was conducted from December 2023 to September 2024 at seven day care centers in Japan.

### 2.2. Participants

Consent to cooperate was obtained from seven collaborating day care centers out of the nine centers approached. Subsequently, 67 participants were recruited through convenience sampling.

The inclusion criteria were as follows: (1) receiving day care service under long‐term care insurance; (2) participating in work‐related activities for at least 6 months; and (3) diagnosis of dementia or MCI, or Mini‐Mental State Examination‐Japanese (MMSE‐J) score ≤ 27. Dementia is characterized by a decline from a previously attained level of cognitive functioning, sufficient to interfere with independence in everyday activities [23]. The criteria for MCI are cognitive complaints (by the patient, informant, or physician), objective memory impairment for age, essentially normal functional activities, and absence of dementia [24]. The exclusion criterion was a life event (e.g., personal major injury or illness, death of a spouse or close relative, divorce, or marital separation) in the last 3 months that could affect mental well‐being [25]. In the present study, work‐related activities were defined as activities contributing to others and the community performed voluntarily as part of the social participation support programs of the day care centers.

### 2.3. Ethical Considerations

This study conformed to the Declaration of Helsinki principles and was approved by the Health Sciences Ethics Committee of Kobe University Graduate School (Approval Number: 1214). All participants and their families provided written informed consent.

### 2.4. Measures

Demographic data, including age, sex, educational history (years of education), hospitalization history (hospitalization within a year), and cognitive function (measured by the MMSE‐J), were collected. The MMSE‐J used in this study was officially licensed and purchased from Psychological Assessment Resources Inc. In addition, the types of work‐related activities were surveyed.

The Japanese version of the World Health Organization‐Five Well‐Being Index (WHO‐5) [26] was used to assess mental well‐being. The tool contains five statements relating to the participant′s experience over the past 2 weeks, rated using a 6‐point Likert scale ranging from 0 (*at no time*) to 5 (*all of the time*). The total score is calculated on a 0–25 scale, with a score close to 0 indicating a lack of well‐being.

In this study, engagement was defined as the act of being occupied or involved with an external stimulus [20]. Engagement in work‐related activities was quantitatively evaluated using the Assessment of Quality of Activities (A‐QOA) [27]. The A‐QOA is an observational assessment tool designed to evaluate a person′s engagement in an activity based on verbal and emotional expression and social communication. It was developed by occupational therapists to find meaningful activities for clients who experience difficulty in expressing their intentions and emotions because of cognitive impairment. The reliability [28] and validity [29, 30] have been verified. The A‐QOA comprises 21 items. Each item is assessed on a 1–4 scale (1 = *not observed*, 2 = *observed to a limited or questionable extent*, 3 = *observed*, and 4 = *observed as a strong or exceptional tendency*) based on the frequency and intensity of each observed item, with a high score indicating more positive reactions to the activity. The AqoaPro software converts results obtained from the ordinal values into continuous variables or probit values, which are adjusted to a normal distribution with the mean and standard deviation (SD) set to 2.5 and 1.0, respectively. In this study, three certified evaluators (occupational therapists with 9–21 years of experience), including the authors, who were trained in the A‐QOA, observed and evaluated the participants from the beginning to the end of their work‐related activities.

Individual factors related to engagement [20, 31] were assessed, including mental well‐being [32], sex, age group, years of education, number of comorbidities, dementia severity, frailty [33], and activities of daily living (ADL) measured by the Tokyo Metropolitan Institute of Gerontology Index of Competence (TMIG‐IC) [34]. Social limitations (due to vision loss, hearing loss, and physical pain) and the consistency of their prior experience with the work‐related activities in which they have participated were also surveyed. As stimulus factors [31], the time spent participating in work‐related activities (time/session) and frequency of participation (sessions/week) were surveyed. As environmental factors [20], the activity location, activity place (indoor or outdoor), and number of people in the group were surveyed.

### 2.5. Statistical Analysis

The survey items were tested for normality using the Shapiro–Wilk test, and their means (SD) and medians (interquartile range [IQR]) or number and proportion of participants (number [percentage]) were calculated.

The severity of dementia was operationally classified based on research by Perneczky et al. [35] and Sugishita et al. [36]: MMSE‐J scores 24–27 as MCI, 21–23 as mild dementia, 11–20 as moderate dementia, and 0–10 as severe dementia. Social limitations due to vision loss, hearing loss, and physical pain were surveyed by asking, “Are there any activities you cannot do because of these factors?” The responses were recorded on a 4‐point scale (*not applicable*, *not quite applicable*, *somewhat applicable*, and *very applicable*) and classified into two groups: *not applicable or not quite applicable* and *somewhat applicable or very applicable*. Participants who met at least three of the five Japanese versions of the Cardiovascular Health Study [33] criteria were considered to have frailty. Frequency of participation in work‐related activities was classified into three groups: ≤ 1, 2–3, and ≥ 4 sessions/week. Participation time/session was classified as ≤ 1 h or ≥ 2 h.

The correlation between the A‐QOA and WHO‐5 scores was evaluated using Spearman′s rank correlation coefficient (*ρ*). Multiple regression analysis was conducted using the A‐QOA as the dependent variable. As individual, stimulus, and environmental factors related to engagement, the WHO‐5, sex, age group, years of education, number of comorbidities, MMSE‐J‐graded severity of dementia, frailty, TMIG‐IC, social limitations (due to vision loss, hearing loss, and physical pain), consistency of prior experience with work‐related activities in which they have participated, time spent and frequency of participation in work‐related activities, activity location, activity place (indoor or outdoor), and number of people in the group were included as independent variables and selected using the stepwise Bayesian Information Criterion. The variance inflation factor (VIF) was also calculated for these variables.

All statistical analyses were performed using EZR Version 1.54 (Saitama Medical Center, Jichi Medical University, Saitama, Japan) [37], and *p* < 0.05 was considered statistically significant. The required sample sizes to detect Spearman′s *ρ* with *α* = 0.05 and a statistical power of 0.9 were estimated to be 67 for *ρ* = 0.4, 43 for *ρ* = 0.5, and 29 for *ρ* = 0.6.

## 3. Results

Tables 1 and 2 present the characteristics of participants and work‐related activities, respectively. A total of 63 participants were included in the final analysis. Of the 67 individuals who were recruited and provided informed consent, four were hospitalized before the data collection date and were therefore excluded from the analysis. The median age was 83 years (IQR: 61, 94). Among the participants, 25.4% were male and 74.6% were female. The median score on the MMSE‐J was 19.0 points (IQR: 4, 27).

**Table 1:** Participant characteristics.

	All
	*n* = 63
Age, years, median (IQR)	83.00 (61.00, 94.00)
Age group, years, *n* (%)	
60–69	4 (6.3)
70–79	13 (20.6)
80–89	36 (57.1)
90–99	10 (15.9)
Sex, *n* (%)	
Male	16 (25.4)
Female	47 (74.6)
Education, years, median (IQR)	12.00 (8.00, 18.00)
Hospitalization within 1 year, *n* (%)	
Yes	5 (7.9)
No	58 (92.1)
MMSE‐J score, median (IQR)	19.00 (4.00, 27.00)
MMSE‐J class, *n* (%)	
27–24 (MCI)	18 (28.6)
23–21 (mild)	8 (12.7)
20–11 (moderate)	31 (49.2)
10–0 (severe)	6 (9.5)
WHO‐5 score, median (IQR)	18.00 (11.00, 25.00)
A‐QOA, mean (SD)	2.93 (0.68)
A‐QOA, *n* (%)	
> 2.5 probit	41 (65.1)
< 2.5 probit	22 (34.9)
Comorbidity, median (IQR)	2.00 (0.00, 7.00)
Hearing loss, *n* (%)	
Not applicable	47 (74.6)
Not quite applicable	5 (7.9)
Somewhat applicable	6 (9.5)
Very applicable	5 (7.9)
Vision loss, *n* (%)	
Not applicable	37 (58.7)
Not quite applicable	16 (25.4)
Somewhat applicable	7 (11.1)
Very applicable	3 (4.8)
Physical pain, *n* (%)	
Not applicable	35 (55.6)
Not quite applicable	10 (15.9)
Somewhat applicable	17 (27.0)
Very applicable	1 (1.6)
Frailty, *n* (%)	
Nonfrail	50 (79.4)
Frail	13 (20.6)
TMIG‐IC, mean (SD)	5.43 (3.32)
Prior experience, *n* (%)	
Consistency	35 (55.56)
Nonconsistency	28 (44.44)

Abbreviations: A‐QOA, Assessment of Quality of Activities; IQR, interquartile range; MCI, mild cognitive impairment; MMSE‐J, Mini‐Mental State Examination‐Japanese; SD, standard deviation; TMIG‐IC, Tokyo Metropolitan Institute of Gerontology Index of Competence; WHO‐5, World Health Organization‐Five Well‐Being Index (Japanese version).

**Table 2:** Characteristics of work‐related activities.

	*n* = 63 (seven facilities)
Type of activity	Wrapping toilet paper, pasting shoji (paper sliding doors), sashiko (stitching), threading drawstring, making thread for sakiori (weaving), preparations for the children′s cafeteria and coffee shop, festival preparations, car washing, posting, leaflet insertion and distribution, cleaning, field work, weeding, and handbell practice (for performances at facilities)
Hours/session, median (IQR)	1.00 (0.40, 3.00)
Sessions/week, median (IQR)	1.00 (0.10, 5.00)
Place, *n* (%)	
Indoor	43 (68.3)
Outdoor	20 (31.7)
Number of people in the group, median (IQR)	5.00 (1.00, 10.00)

Abbreviation: IQR, interquartile range.

### 3.1. Relationship Between Engagement in Work‐Related Activities and Mental Well‐Being

**Figure 1:**
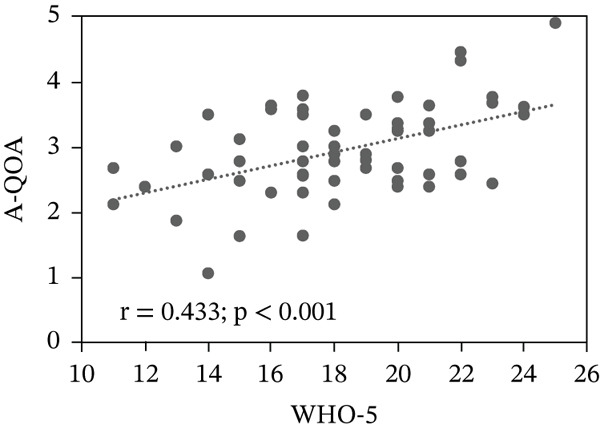
Correlation between engagement in work‐related activities and mental well‐being. Ordinal values of the A‐QOA converted to continuous variables (probit values) using the AqoaPro software application. Probit values were adjusted to a normal distribution with a mean of 2.5 and a standard deviation of 1.0. Note: A‐QOA, Assessment of Quality of Activities.

The A‐QOA and WHO‐5 had a Spearman′s *ρ* = 0.433 (*p* < 0.001), confirming a positive correlation between engagement and mental well‐being (Figure 1).

### 3.2. Factors Influencing Engagement in Work‐Related Activities

The multiple regression analysis results are presented in Table 3. We observed a significant association between the A‐QOA and WHO‐5 (*β* = 0.480, 95% confidence interval [CI] [0.054, 0.143], *p* < 0.001), frequency of participation (*β* = 0.337, 95% CI [0.118, 0.569], *p* = 0.003), and social limitations due to hearing loss (*β* = 0.286, 95% CI [0.120, 0.895], *p* = 0.011). The adjusted *R* ^2^ for this model was 0.358 (*p* < 0.001). There was no missing data for any of the variables included in the analysis.

**Table 3:** Factors related to engagement in work‐related activities in people with dementia (*n* = 63).

Independent variable	*β*	*t*	SE	95% CI	*p* value	VIF
WHO‐5	0.480	4.461	0.022	(0.054, 0.143)	< 0.001	1.117
Frequency	0.337	3.053	0.113	(0.118, 0.569)	0.003	1.176
Hearing loss	0.286	2.621	0.194	(0.120, 0.895)	0.011	1.147
Multiple *R* ^2^: 0.389					< 0.001	
Adjusted *R* ^2^: 0.358						

*Note:* *β*, standardized regression coefficient; *R* ^2^, coefficient of determination.

Abbreviations: CI, confidence interval; SE, standard error; *t*, *t*‐statistic; VIF, variance inflation factor; WHO‐5, World Health Organization‐Five Well‐Being Index (Japanese version).

## 4. Discussion

This study investigated the association between engagement in work‐related activities and mental well‐being in people with dementia or MCI and clarified the factors that influence engagement in work‐related activities. Greater engagement in work‐related activities was associated with better mental well‐being, higher frequency of participation, and social limitations due to hearing loss.

The results revealed an association between greater engagement in work‐related activities and better mental well‐being in people with dementia or MCI, in concurrence with that reported in previous studies [38, 39]. These findings suggest that increasing engagement may be important in improving mental well‐being through participation in work‐related activities.

The results revealed an association between a higher frequency of participation and greater engagement in work‐related activities. de Werd et al. [40] reported that people with dementia are still able to acquire meaningful skills and engage in worthwhile activities. An increased frequency of participation is linked to repeated participation and enables habitual and smooth performance, as well as encouraging interaction through relationships with coparticipants. Therefore, these findings suggest that increasing the frequency of participation may be important for enhancing engagement in work‐related activities among people with dementia or MCI. People with dementia have a reduced stress threshold and are unable to cope with environmental stressors, resulting in the onset of peripheral symptoms [41]. An increased frequency of participation in work‐related activities reduces the burden of each session, leading to reduced environmental stressors and ultimately reduced stress.

In this study, social limitations due to hearing loss were associated with a robust engagement. In contrast to the findings of the present study, Cohen‐Mansfield et al.′s [31] survey on engagement in personalized recreational activities among nursing home residents with dementia reported weak engagement among those with hearing loss. Additionally, people with dementia and hearing loss demonstrated weak engagement in ADL [31], with some also experiencing loneliness [42]. In contrast, in the present study, which involved specific work‐related activities, participants with hearing loss needed longer or more frequent contact with support staff than those without, requiring individualized explanations and adjustments. Moreover, work‐related activities frequently involve groups of two or more people, and in the absence of nearby support staff, participants can compensate with visual cues from others performing similar activities. Hence, work‐related activities are meaningful activities of a higher value than ADL, which could increase participants′ engagement.

Several participants in this study had a robust engagement in work‐related activities. You and Miura [43] reported that older people who require care exhibit significantly stronger engagement in work‐related activities than in recreational activities. Nursing homes are generally care providers, with people with dementia as service consumers [44]. Participation in work‐related activities transforms patients from “receiving support” to “providing support,” which may be linked with robust engagement [45, 46].

## 5. Limitations

This study has some limitations. First, it is a cross‐sectional survey; therefore, the causal relationship between engagement in work‐related activities and the factors selected in the study′s multiple regression analysis cannot be determined. Second, the participants were selected by convenience sampling, thus leading to possible biases, and they may not represent the general population. Third, the number of variables that could be included in the multiple regression analysis was limited by the insufficient participant numbers; hence, some important factors may have been overlooked. Fourth, previous research has indicated that stimuli from light and sound, and the number of support workers involved, may influence engagement; however, we only partially investigated these factors. In addition, information on hearing aid use, personality traits, work attitudes, compensatory strategies, and other potential confounding factors was not collected, which limits the interpretation of the observed association between A‐QOA and social limitations due to hearing loss. Future studies using longitudinal designs with larger samples based on sample size calculations and incorporating more comprehensive assessments of factors related to engagement should augment the current evidence.

## 6. Conclusions

This study found that better mental well‐being, higher frequency of participation, and social limitations due to hearing loss were associated with greater engagement in work‐related activities. Future studies should provide further evidence considering the present study′s limitations, identify suitable methods of personalized support, and establish effective methods for implementing work‐related activities that improve the mental well‐being of people with dementia or MCI.

## Funding

This study was funded by the Japan Science and Technology Agency SPRING (JPMJSP2148) and the Sugiura Memorial Foundation.

## Conflicts of Interest

The authors declare no conflicts of interest.

## Data Availability

The data that support the findings of this study are available from the corresponding author upon reasonable request.
